# Seasonal calibration of the end-cretaceous Chicxulub impact event

**DOI:** 10.1038/s41598-021-03232-9

**Published:** 2021-12-08

**Authors:** Robert A. DePalma, Anton A. Oleinik, Loren P. Gurche, David A. Burnham, Jeremy J. Klingler, Curtis J. McKinney, Frederick P. Cichocki, Peter L. Larson, Victoria M. Egerton, Roy A. Wogelius, Nicholas P. Edwards, Uwe Bergmann, Phillip L. Manning

**Affiliations:** 1 Department of Earth and Environmental Sciences, University of Manchester, Manchester, M13 9PL UK; 2 Department of Geosciences, Florida Atlantic University, Boca Raton, FL 33431 USA; 3 Biodiversity Institute, University of Kansas, Lawrence, KS 66045 USA; 4 Department of Biological Sciences, Southwestern Oklahoma State University, Weatherford, OK 73096 USA; 5 Department of Geology, Miami-Dade College, Miami, FL 33132 USA; 6 Adjunct Curator of Vertebrates, Maine State Museum, Augusta, ME 04330 USA; 7 Black Hills Institute of Geological Research, Hill City, SD 57745 USA; 8 Stanford Synchrotron Radiation Lightsource, SLAC National Accelerator Laboratory, Menlo Park, CA USA; 9 Department of Physics, University of Wisconsin- Madison, 2320 Chamberlin Hall, 1150 University Avenue, Madison, WI USA

**Keywords:** Evolution, Environmental sciences, Natural hazards, Astronomy and planetary science

## Abstract

The end-Cretaceous Chicxulub impact triggered Earth’s last mass-extinction, extinguishing ~ 75% of species diversity and facilitating a global ecological shift to mammal-dominated biomes. Temporal details of the impact event on a fine scale (hour-to-day), important to understanding the early trajectory of mass-extinction, have largely eluded previous studies. This study employs histological and histo-isotopic analyses of fossil fish that were coeval with a unique impact-triggered mass-death assemblage from the Cretaceous-Paleogene (KPg) boundary in North Dakota (USA). Patterns of growth history, including periodicity of ẟ^18^O and ẟ^13^C and growth band morphology, plus corroborating data from fish ontogeny and seasonal insect behavior, reveal that the impact occurred during boreal Spring/Summer, shortly after the spawning season for fish and most continental taxa. The severity and taxonomic symmetry of response to global natural hazards are influenced by the season during which they occur, suggesting that post-impact perturbations could have exerted a selective force that was exacerbated by seasonal timing. Data from this study can also provide vital hindsight into patterns of extant biotic response to global-scale hazards that are relevant to both current and future biomes.

## Introduction

The Chicxulub impact is widely regarded as the primary driver for the Cretaceous-Paleogene (KPg) mass extinction^1–3^ and launched multiple long-term, planet-wide impacts for life on Earth. A globally distributed Iridium-rich clay at the KPg boundary, constituting the re-accretion of impact fallout, was one of the first indicators of a massive extra-terrestrial impact^1^. Impact-triggered tsunami deposits have been reported in nearshore-marine and continental shelf deposits around the Gulf of Mexico and the Caribbean, < 1000 km from the Chicxulub crater^4–6^, and evidence of seismically induced surges ~ 3000 km from the crater were recently documented at the Tanis (North Dakota, USA) KPg mass-death assemblage^7^. Extreme, long-term global climatic shifts including a prolonged multi-year dark post-impact winter, resulting from infusion of CO_2_ and SO_2_ admixed with soot and atmospheric dust^1,8,9^, are considered the primary agents of critical terrestrial and marine ecological collapse^10^. Impact-related environmental perturbations ultimately resulted in Earth’s last known mass-extinction event^3^.

The damaging effect of the multiple resulting causal triggers of extinction can vary depending on the time of year, therefore identifying the season for the Chicxulub impact event may be a crucial key to assessing the initial biotic stresses and also better help resolve their global effects. Several prior studies, including Wolfe^11^ and others, have sought to reconstruct the time of year for Chicxulub impact, however thus far no well-supported consensus has been reached. Such an endeavour is dependent on examining a time scale much finer than typically identifiable in the stratigraphic and fossil record. In this study, we determine the time of year for the Chicxulub impact via a multi-year investigation that began in 2014, which has focused on histological observations of multiple coeval vertebrate skeletons entombed by an impact-triggered surge.

The study locality (Tanis) is located in the uppermost strata of the Hell Creek Formation of North Dakota (U.S.A.) near the predicted terminal Cretaceous paleoshoreline of the Western Interior Seaway (WIS) [SUP MAT 1]. The site preserves a rapidly emplaced ejecta-bearing sedimentary package generated by an impact-triggered seiche. The Tanis site possesses a highly constrained sedimentological chronology that is uniquely suited to examine the immediate post-impact events in a highly refined time-scale^7^. The Tanis Event-deposit preceded the iridium-rich dust-sized fallout and was emplaced exclusively within the period of coarse ejecta accretion, which began in the study region ~ 13 min after impact and lasted for ~ 1 to 2 hours^7^. The massive water surge, possibly originating from the nearby WIS, entombed the remains of autochthonous freshwater fish, turtles, reptiles, dinosaurs, and plants, mixed with allochthonous marine organisms including fish, ammonites, dinoflagellates, foraminifera, and marine reptiles, all intimately associated with impact ejecta emplaced via primary deposition. The sediment package is capped and temporally constrained by an iridium-rich clay layer (tonstein) that typifies the KPg boundary in the Western Interior [SUP MAT 2].

Tanis is characterized by a condensed synchronous thanatocoenosis (death-assemblage) that is thus far the only known preserved grouping of Chicxulub ejecta and articulated macro-organisms that died at the KPg boundary^7^. Acipenseriform fish (paddlefish and sturgeon), with sizes representing multiple growth stages from young-of-the-year (YOY) to mature adult^12–16^, are the most abundant vertebrates observed at the Tanis Konservat Lagerstätte (death assemblage characterized by atypically well preserved and articulated body fossils). The smallest Acipenseriformes at Tanis (< 16 cm fork length) fall below the expected length of yearling extant acipenseriform taxa^12–16^, and we interpret that they died during sub-yearling ontogeny (i.e. YOY).

The vertebrate skeleton relies upon a series of trace-metal cofactors that correlate with different stages of bone growth that can be compared to ontogenetic stage and can be quantified through synchrotron-based imaging^17–21^. Synchrotron Rapid Scanning X-ray Fluorescence (SRS-XRF) imaging^22,23^ was performed to better visualize osteology and help determine the degree of ossification of fossil Acipenseriformes based on trace metal signatures related to growth^18^. Histological examination in this study focused on 1) morphology of growth bands and lines of arrested growth (LAGs) in fish bone using ordinary light microscopy, and 2) ẟ^18^O and ẟ^13^C analysis of the growth bands. As a result, we found evidence for seasonal changes in growth and physiological condition. These analyses were complemented with data on body sizes of YOY fishes, as well as indications of seasonally dependent insect activity.

## Results and discussion

### Histology and isotopic analysis

Bone growth corresponds directly with seasonal physiological condition and overall health, with one unit produced each year, terminating at a line of arrested growth (LAG)^13,24,25^. Variable bone growth within the year is represented by a couplet of two bands subdividing the annual unit^13,24,25^. A dark layer of bone, corresponding with Spring and Summer months, arises from increased food consumption and higher metabolic rate/growth; a light band less populated with osteons is apposed during the Fall and Winter months^13,26^.

The annual temperature gradient of the latest Cretaceous U.S. Western Interior, which fluctuated by ~ 13°C^27,28^, was sufficient to record seasonal variation in osseous lines of arrested growth (LAGs) and in woody-stemmed flora^28,29^, and aided in the legibility of our histological samples. Well-resolved successive annual growth packages comprised of rapid- and slow-growth separated by LAGs were observed in all Acipenseriformes sampled from Tanis, cumulatively recording the last few decades of growth history preceding the Chicxulub impact [SUP MAT 3–8]. Bone growth in the Tanis fishes was cut short, prior to the Fall-Winter period of slower growth (Fig. 2C, D). This pattern suggests that death occurred during peak growing season, which is likely to have been the Spring and Summer months^13–15^, broadly agreeing with the earlier study made by Wolfe^11^.

Food uptake by Acipenseriformes is episodic, generally highest in Spring and Summer^12,13,30–33^, during which a higher ^13^C/^12^C ratio results from higher uptake of ^13^C^34–36^. The fossil fishes from Tanis exhibit a repeated pattern in the carbon isotopic ratios, alternating between lighter and heavier ẟ^13^C (Fig. 2B; SUP MAT 9, 10). The lightest carbon values (lowest productivity) correlate with lines of arrested growth, while the heaviest values (peak productivity) correlate with the dark rapid-growth zones of each annual unit. Based on modern behavioral patterns, the light Tanis carbon values correlate with Fall to Winter months while the heaviest values correlate with the peak productivity of Spring to Summer^12,13,34–36^. Carbon isotope data from the outermost (terminal) layer of bone correlates with the heaviest values recorded in earlier bands, indicating that death occurred during an interval of heightened productivity that aligns with northern latitude Spring/Summer.

**Figure 1:**
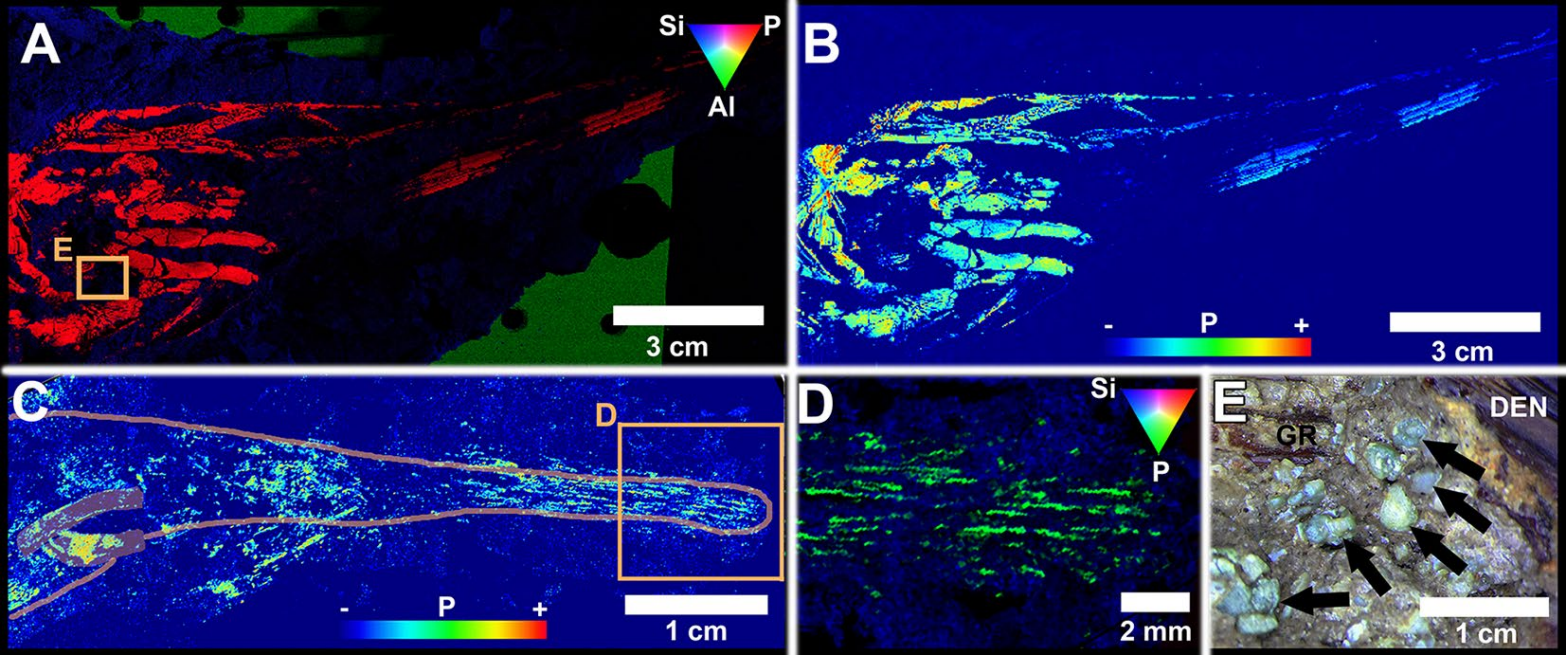
SRS-XRF elemental mapping of two polyodontids from Tanis [Draft image; replace with actual figure in editing phase]. The skull of a yearling fish in lateral view (FAU.DGS.ND.725.32.T) is shown as (**A**) a tricolor plot (red = phosphorus; blue = silicon; green = aluminium) and (**B**) a heat map for phosphorus, showing high definition of well ossified articulated skull elements. The skull of a sub-yearling polyodontid (FAU.DGS.ND.711.14.T) exhibits underdeveloped osseous anatomy characteristic of an earlier ontogenetic stage (**C**), however still possesses well-recognizable rostral elements (**D**). (Tricolor plot in D: green = phosphorus; blue = silicon; red channel muted). (**E**), A magnified view of the skull in (**A**, **B**) seen under normal light shows multiple impact spherules (arrows) near the gill rakers (GR = gill rakers, DEN = dentary).

The trace-metal cofactors that the vertebrate skeleton relies upon at different growth stages can also be compared to discrete growth stages, this determined through synchrotron-based imaging^17–21^. Synchrotron Rapid Scanning X-ray Flourescence (SRS-XRF) imaging^22,23^ has been successfully used to help visualize osteology and also determine the degree of ossification of fossil bone based on trace metal signatures that relate to growth^18^. The SRS-XRF scans revealed the weakly ossified and immature state of the juvenile fish when compared to adult specimens^12^ (Fig. 1), and better resolved the nearly pristine articulated skeletal anatomy. The skull and embedding matrix of a yearling fish in lateral view (FAU.DGS.ND.725.32.T) in Fig. 1(A) is distinguished here by three elements; phosphorus (red), silicon (blue) and aluminium (green). The phosphorus (blue) in the hydroxyapatite skeleton is clearly differentiated against the surrounding silica-rich mudstone matrix (Fig. 1A). A ‘heat map’ for phosphorus shows the relative concentrations of this element in well ossified articulated skull elements (Fig. 1B). However, the skull of a sub-yearling polyodontid (FAU.DGS.ND.711.14.T) exhibits underdeveloped osseous anatomy characteristic of an earlier ontogenetic stage (Fig. 1C), but still possesses well-recognizable rostral elements (Fig. 1D). Many of the fish at Tanis (~ 50% of those examined thus far) contained ejecta spherules lodged in their gill rakers (e.g. ref. 7, Fig. 1 D), aspirated passively from the water column during ejecta accretion^7^. This demonstrates that the fish were alive at the time of coarse ejecta arrival and died during the violent surge and were entombed by seiche sediments, prior to the final emplacement of the distinctive iridium-rich capping clay.

**Figure 2:**
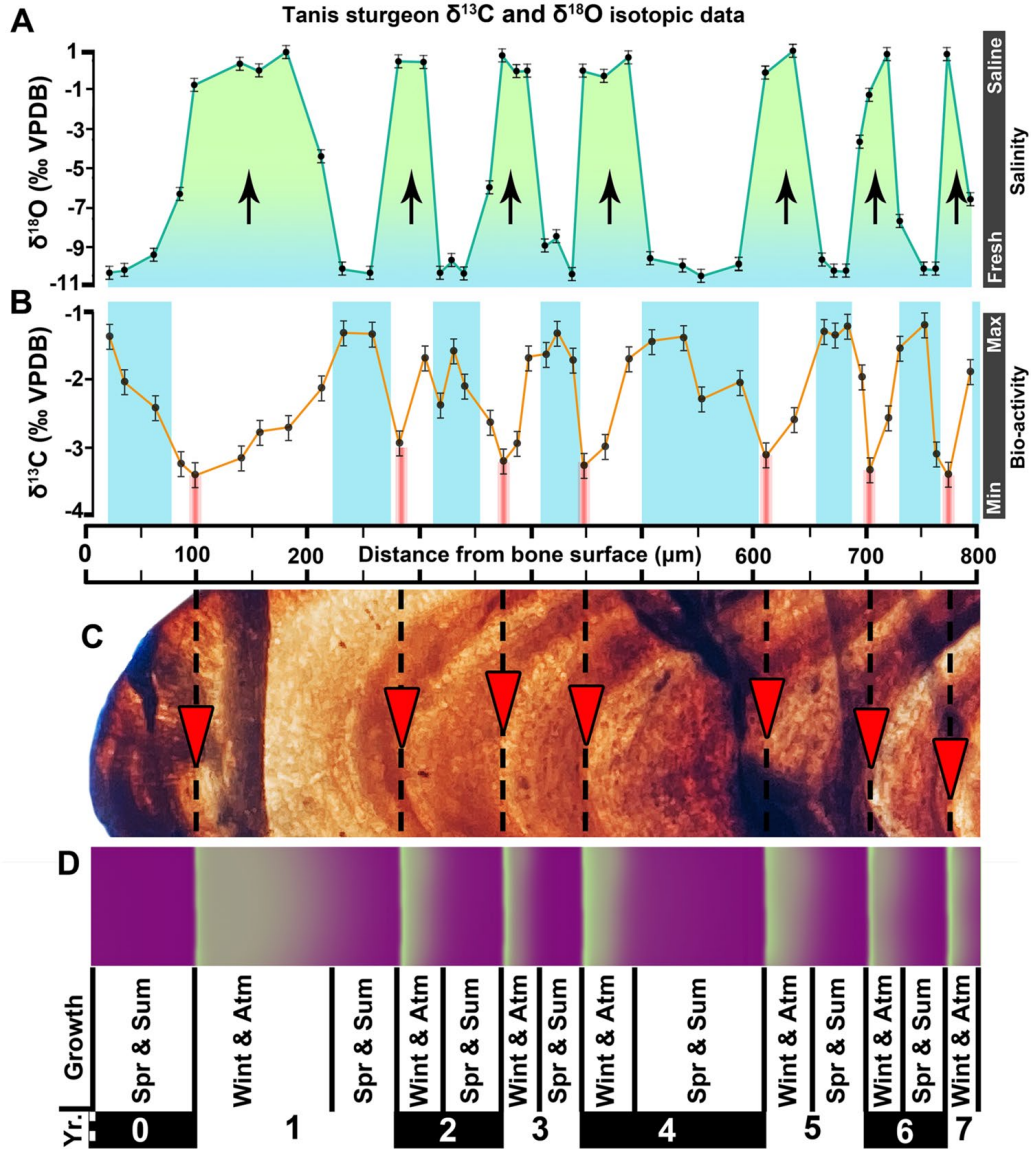
Bone growth patterns reflected in isotopic and histological data. (**A**) *δ*^18^O data from a sturgeon pectoral fin spike (FAU.DGS.ND.755.57.T) shows a repeated fluctuation between saline (arrows) and freshwater living conditions. The freshwater zones of bone growth correlate with peak times in biological productivity (blue bands in [**B**]), while the saline zones correlate with lowest productivity that are histologically consistent with winter months^13,24–26^. (**B**) *δ*^18^C values for incremental samples from the same thick section, taken 1 mm adjacent to the samples in (**A**), showing multi-year fluctuations that inversely relate to the oxygen data. (**C**) a photomicrograph of the same fin spike, showing fluctuations in annual growth history that correlate with the isotopic data. Red arrows point to annual lines of arrested growth (LAGs) deposited during winter. (**D**) an idealized schematic of the histological patterns in (**C**), with subdivisions of growth bands labelled according to their season of growth^13,24–26^.

Oxygen stable isotopic ratios provide additional evidence that helps in the interpretation of life-history of the Tanis sturgeon. Incremental histological sampling of bone tissue revealed a regular periodicity between light and heavy ẟ^18^O values that were out of phase with the carbon data (Fig. 2). The lightest values, which correlate with the heaviest values for carbon, are consistent with a freshwater provenance, however the heavier values (> -5‰) indicate a saline environment^7,37^. The heaviest values (-1 to 1‰) are consistent with fully marine allochthonous fossils found at Tanis, including ammonite shell material and some selachian teeth^7^, that comprise our closest approximation to terminal Cretaceous WIS water chemistry since no geologic record of the seaway from that time exists. At the time of mortality, the Tanis sturgeon displayed light ẟ^18^O values consistent with the lowest values for the prior years. Coupled with the carbon isotope data, this indicates that death occurred during Spring or Summer months. While processed ẟ^18^O data can also be employed to infer variation in temperature, we feel that our data better supports concentration ranges for salinity, particularly when compared with the Polyodontidae. Oxygen isotopic data for the Tanis polyodontids (paddlefish), whose modern counterparts are entirely freshwater fishes^12^, did not exhibit ẟ^18^O ratios outside of the freshwater range, even though the polyodontid ẟ^18^C periodicity correlated with the sturgeon [SUP MAT 11, 12].

The salinity data suggests that Cretaceous sturgeon were diadromous (analogous to their extant relatives), capable of experiencing a marine environment during the Winter months of each year (correlating with the lightest ẟ^18^C values), prior to returning to fresh water in the Spring/Summer^31–33,38^. Anadromy is the most common form of diadromous lifestyle, such that extant sturgeon migrate into freshwater during spawning season, and then back out to sea or estuary. A smaller proportion are catadromous, living in freshwater and migrating to sea for spawning. Brief ventures into salt or fresh water may also occur sporadically throughout the year. Because the regular periodicity of the ẟ^18^C is of the same order as the yearly bone growth pattern, the Tanis sturgeon seem to have been strictly anadromous. This evidence for migratory behaviour is an important datum for Mesozoic sturgeon, the early evolution and life habits of which are very poorly known^12^.

### Independent verification: ontogenetic calibration and insect behavior

**Figure 3:**
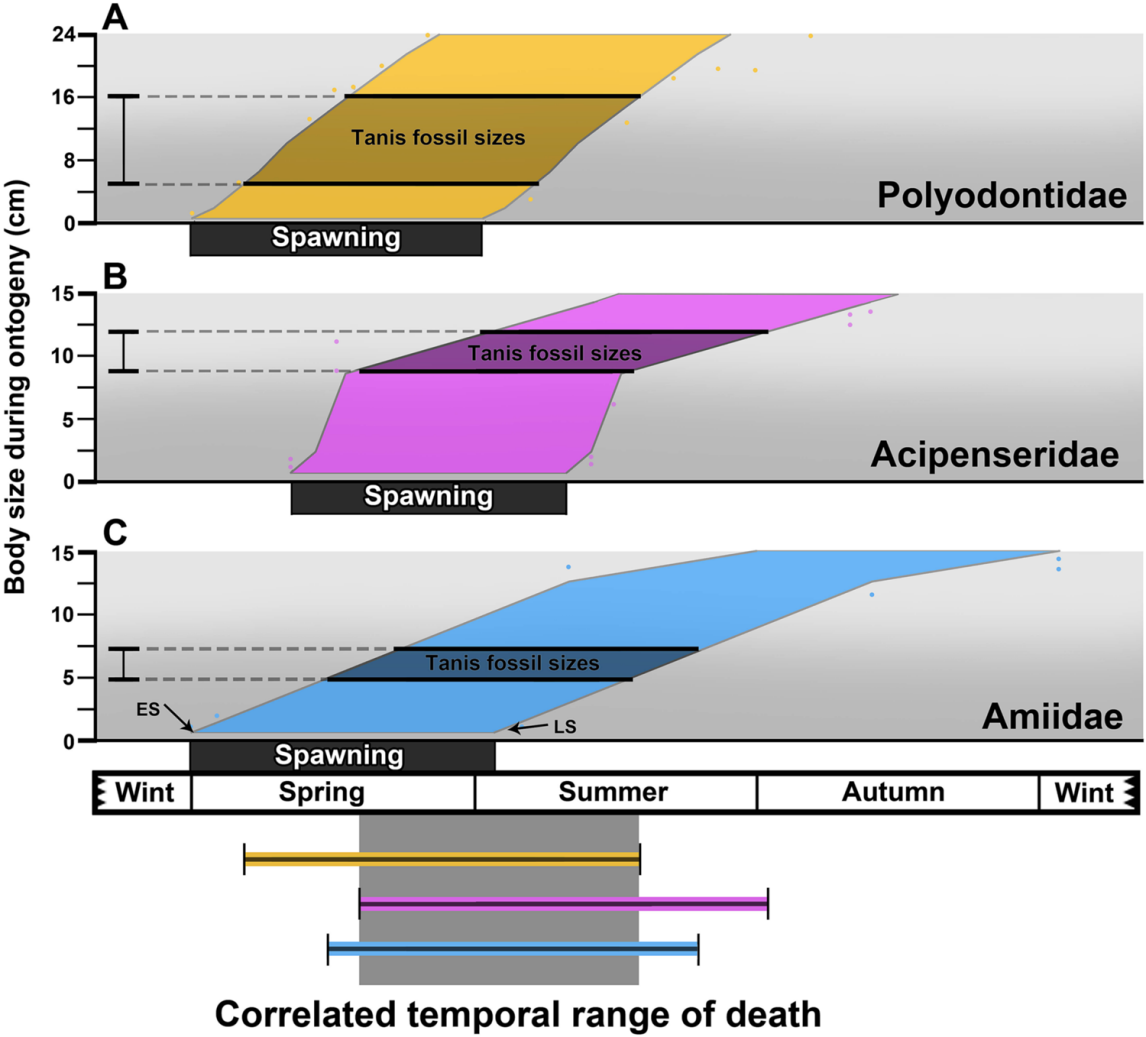
Estimated chronology of death for Tanis sub-yearling fish. A-C, compiled ranges of body sizes for modern fish taxa (Polyodontidae, Acipenseridae, Amiidae)^12,14–16,30,31,39,43–45^ relative to time span after spawning. Bracketing this growth data between early spawning (ES) and late spawning (LS) starting points gives a full spectrum of possible seasonal end-points relative to a given body size (shaded region). The Tanis specimens broadly ranged between early-mid Spring and late Summer. The correlated overlapping regions refined this further, identifying the time of death as late Spring to mid-Summer.

The body sizes of YOY fish at Tanis enabled us to make a reasonable estimate of the elapsed time between hatching and death. A wide range of YOY body sizes relative to growth rate were compiled from the literature for modern Acipenseriformes^12,14–16,30,31,40^, comprising a sufficiently broad spectrum of slow and rapid growth to encompass the imprecisely established ranges of the ancestral Cretaceous taxa. The body sizes of YOY fish from Tanis were compared with these ranges to estimate the time since hatching (Fig. 3). A maximum temporal range was established by using the earliest and latest typical spawning times for modern Acipenseriformes, which spans from Spring to Summer^30–33,38^. The less-frequent dual annual spawning that occurs with some modern taxa (e.g. refs. ^41,42^) was not considered, as it is not supported by the YOY body sizes seen at Tanis. The acipenseriform microtemporal bio-calibration was complemented by a similar examination of YOY amiid fish from the site^43–45^. As a result, this data suggests that the death assemblage occurred between mid-Spring and late Summer, in agreement with the histological and histo-isotopic data.

Insect activity that can reliably be linked with seasonal behavior further supports the histological, growth stage, and isotopic data. Leaf mining is a widespread behavioral strategy that has been independently adopted by a variety of unrelated insect taxa, primarily Lepidoptera (moths), Symphyta (sawflies), and Diptera (true flies)^46^. In all cases, leaf mining follows a regular pattern, beginning with the hatching of larvae typically in the Spring. The larvae quickly seek out leaves and burrow inside, excavating a taxonomically informative pattern as they consume leaf tissue. The process of leaf mining maximizes during the peak growing season, typically Spring and Summer months, and is not typically associated with Winter or Fall^47–49^. At Tanis, leaf mining damage was abundant and observed on ~ 40% of angiosperm leaves that were contemporary with the depositional event (SUP MAT 13), some of which were still attached to branches. Nearly all leaf mines exhibited single-tunnel serpentine morphology that followed the major veins and contained no obvious frass tubes, however detailed treatment of the leaf miner taxonomy or mine morphology are outside the scope of this study. The abundance of leaf mining observed in the Tanis angiosperm leaves indicates that the time of deposition was aligned with active leaf mining. Modern examples indicate that the mining activity at Tanis was most likely during the Spring or Summer, less likely for Autumn, and not possible during Winter^47–49^. Given the rapid emplacement and high temporal fidelity of the Tanis sedimentary package that contains leaf fossils, along with their complete and often attachment to branches, indicates that they were contemporary to the moment that the KPg impact occurred. It is also highly improbable that such delicate fossils of leaves were capable of being reworked from earlier deposits into the Tanis depositional event.

Additional seasonally aligned insect activity at Tanis is represented through mayflies (SUP MAT 14, 15). The annual periodicity of synchronized adult mayfly emergence is constrained to a very short time span^50–52^, and therefore is a reliable tool for temporal constraint. Body fossils of multiple adult mayflies occur as compression fossils in the fine-grained silt in upper Unit 2 and uppermost Unit 1 of the Event-deposit (SUP MAT 15). Mayfly burrow casts, some of which preserve the remains of larval mayflies, have also been found excavated into the wood of large (~ 20–30 cm diameter) tree trunks and aligned in dense subparallel groupings that follow the grain of the wood (presumably following the path of least resistance). The burrows, preserved in full relief as matrix infillings, occur exclusively in wood that had previously died, i.e. exhibited no attached branches with leaves or other evidence that they were fresh at the time of burial. Extant mayflies exhibit highly constrained annual behaviour, beginning their life cycle during the Spring spawning period, at which time eggs are deposited in a freshwater environment^50–52^. Larvae, living on the bottom or in U-shaped burrows excavated into soft substrate, mature over a period of months to years. The final adult moult and emergence occur *en masse* during a very short (< several week) time span that typically occurs in the latest Spring and Summer, between April and July^50–52^. Adults live subaerially only for a period of hours to days before dying in large groups. The adult mayfly body fossils at Tanis therefore indicate that the depositional event occurred during a short time span in late Spring or Summer, after the appearance-window for adult mayflies, and prior to a complete disintegration of their very delicate bodies.

## Conclusions

**Figure 4:**
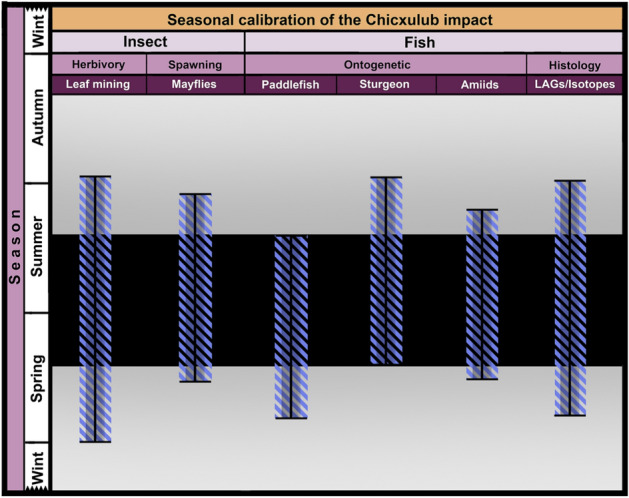
Seasonal calibration of the Chicxulub impact. Correlated ranges of seasonal insect behavior, inferred time of year based on sub-yearling fish fossil size ranges, and histological data (visual and isotopic) provide a refined temporal estimate for the Chicxulub impact event.

Histological and histo-isotopic data from acipenseriform fishes at Tanis, independently supported by seasonally mediated animal behaviour, reveal that the Mesozoic came to an abrupt end in the paleo equivalent of late Spring or Summer in the northern hemisphere, aligned closely with spawning season (Fig. 4). The initial devastating effects of the Chicxulub impact would have been amplified for biota in the northern hemisphere that possessed vulnerabilities inherent to this time span, which was a period of growth and reproduction for many animals and plants^1,3,53^. Mass mortality of young would have been especially disastrous for species that took many years to reach breeding age or bred only under ideal conditions. The rapid onset of impact-triggered effects, such as atmospheric contamination, blockage of sunlight, and rapid shifts in temperature, would have been felt harder in highly seasonal ecologies. It is therefore plausible that this phenomenon predisposed an asymmetric pattern of extinction between the northern and southern hemispheres.

Resolving fine-scale details from the KPg event, such as its seasonal timing, can improve our understanding of the immediate and longer-term effects of the Chicxulub impact on Earth’s biota. The results from this study may facilitate a better understanding of how seasonally mediated vulnerabilities of Cretaceous taxa could have been exacerbated by the impact, leading to enhanced selective pressures that influenced the trajectory of this mass extinction. Ultimately, insight into a globally devastating mass extinction event such as Chicxulub provides the only direct evidence of biotic responses to catastrophic trauma of greater magnitude than what is documented in written history. Thus, it is critical to utilize the hindsight that the fossil record affords us, at such global-scale tipping-points, so that we might better understand how to mitigate for the contemporary extinctions impacting life on Earth.

The specimens and data in this study are accessioned at Florida Atlantic University (FAU) Department of Geosciences, under the catalogue numbers as they appear in this manuscript.

## Materials and methods

### Synchrotron elemental mapping

Synchrotron radiation and the specific capabilities of the beam lines at the Stanford Synchrotron Radiation Lightsource (SSRL) are the only way we can spatially resolve elemental distributions on the scales and concentrations that we were interested in this study. This station uses a continuous rapid-scan system with a scan range of 1000 × 600 mm and a load capacity of up to 25 kg, capable of 25–100 μm resolution elemental XRF mapping and X-ray absorption spectroscopy (XAS) of a wide range of objects. XRF is measured using a four-element Hitachi Vortex ME4 silicon drift detector coupled to a Quantum Detectors Xspress3 multi-channel analyser system. A custom system allows the X-ray spot size to be changed quickly and easily via pinholes ranging from 25 to 100 μm. The instrument is located at wiggler beamline 6–2 which has an energy range of 2.1– 17 keV, creating K emission for elements up to strontium, and L or M emission for all other elements. XAS can also be performed at selected sample positions within the same experiment, allowing for a more detailed chemical characterization of the elements of interest. Furthermore, sparse excitation energy XRF imaging can be performed over a wide range of incident X-ray energies. User friendliness has been emphasized in all stages of the experiment, including versatile sample mounts, He purged chambers for low-Z analyses, and intuitive visualization hardware and software. The station provides analysis capabilities for a wide range of materials and research fields including biological, chemical, environmental and materials science, palaeontology, geology and cultural heritage^54^.

Synchrotron Rapid Scanning-X-ray Fluorescence (SRS-XRF) imaging of Low-Z elements was performed in this study at wiggler beamline 6–2 (SSRL) and experiments were carried out following procedures elaborated by prior studies conducted at the same facility^22,23^. Incident beam energy for the experiments was again set for High-Z elements at 13.5 keV (flux between 10^10^ and 10^11^ photons^-1^), and at 3.15 keV (flux ~ 10^9^ photons^-1^) for Low-Z mapping. Experiments utilized a beam diameter of either 50 or 100 µm, defined by a pinhole. The four-element Hitcahi Vortex ME4 silicon drift detector was used to detect fluoresced X-rays. SRS-XRF data underwent initial processing from raw detector values to visible element distribution maps via the SMATK program (https://www.sams-xrays.com/smak) custom developed by the Stanford Linear Accelerator Center (SLAC).

### Thin sections and light microscopy

In this study, the skull bones of fossil polyodontids (paddlefish) and the pectoral fin spines of fossil acepenserids (sturgeon) provided the most reliable uninterrupted growth record and were preferred over endochondral bone due to heightened reliability of data, legibility of morphology, and precedent set by prior studies^13,24,25,39,55^. A total of 7 fossil adult polyodontids and 12 acipenserids were selected for histological analysis.

All samples were first embedded in epoxy prior to sectioning. The thin and thick sections (~ 30 micron and ~ 250 micron thickness respectively) were cut perpendicular to bone growth and prepared by National Petrographic Services, Houston, Texas. Thick sections were cleaned in an ultrasonic bath prior to collection of analytical samples to minimize potential surface contamination. Initial observations were made with an Optima ZM-160AT dissecting scope. Additional observations were made using an Ernst Leitz Wetzlar light microscope, Olympus BH2, and Leica DM750P in normal, polarized, and cross-polarized light.

### Field work

Field efforts related to this study took place between project initiation in 2014 until summer 2016, with some final work extending into 2017 with field assistance from students Melanie During, Pim Kaskes, and their advisor Jan Smit. Samples were taken only from complete individuals that were conclusively part of the synchronous mass-death assemblage; isolated skeletal elements were avoided. Fossil fish specimens almost exclusively were represented by articulated skeletons closely appressed to each other in entangled masses oriented by flow. Body length measurements and osteo samples were therefore taken from individuals that remained in their contextual matrix block. Stratigraphic data was collected for each sampled fish. Contextual sediment samples or features of interest (e.g. ejecta lenses, sedimentary structures, etc.) were collected for each sampled fish for archived reference.

### Isotope geochemistry

Isotopic analyses were performed by Curtis McKinney, professor of geology, Miami-Dade College, Miami, Florida. Thick-section wafers of Tanis acipenseriform bone were incrementally sampled from core to bone surface with a New Wave Research Micromill using a Brasseler tungsten carbide mill bit along a drill transect perpendicular to the surface of the bone. The drill bit was cleaned after each spot-sample. Samples were analyzed using a Gas Bench II linked to a Thermo Finnigan duel-inlet MAT 253 Stable Isotope Ratio Mass Spectrometer. C and O isotopic ratios were reported according to the VPDB international standard, with precision of ± 0.3‰.

## Supplementary Information

Supplementary Information.
