# Supplementary material for: Seasonal calibration of the end-cretaceous Chicxulub impact event
Source: Sci Rep. 2021 Dec 8;11:23704. doi: 10.1038/s41598-021-03232-9 (PMC8655067; doi:10.1038/s41598-021-03232-9)

Supplemental Materials

SUP MAT 1. **Paleogeographic and depositional setting of the Tanis deposit.** (A), Tanis (*star in insert) within the localized study region (large map) and in context near the western shore of the Western Interior Seaway. (B), Field photos and interpretive overlay of an oblique cross-section of the Event-deposit, which was left as a mud drape on the surface of a paleo river point-bar following a massive inland-directed surge. (C), Graphic showing the Event-deposit and depositional geometry; 1: drape-like Event-deposit; 2: underlying point-bar sandstones; 3: densest concentration of carcasses and bio-debris; 4 and 5: exposures of the KPg tonstein overlying the Event-deposit and adjacent embankment, respectively; 6: exposure of the KPg tonstein at Brook Butte. Image adapted from DePalma et al^7^.


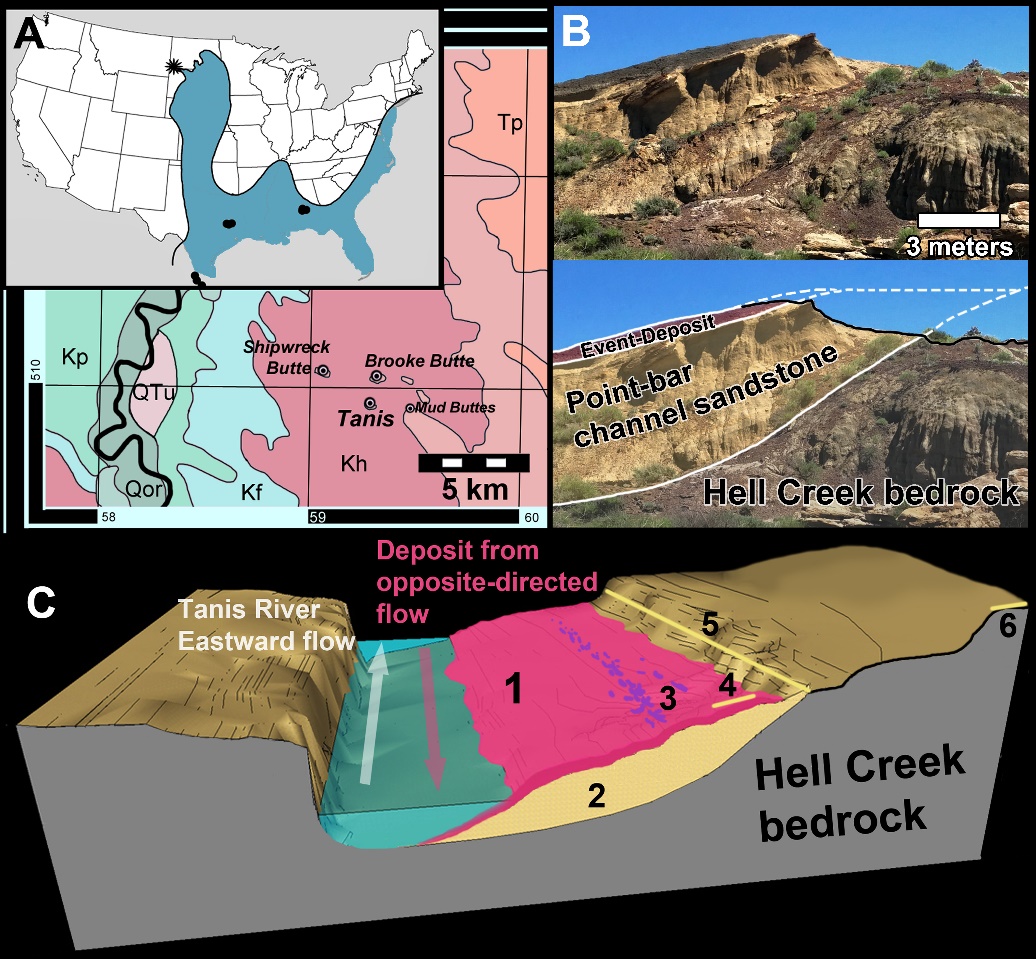


SUP MAT 2. **Stratigraphic and sedimentary log from Tanis.** Stratigraphic occurrence within discrete sedimentary packages identified at site, also marking the temporal location of key elements such as carcass concentration, ejecta, and the overlying KPg iridium anomaly. Image adapted from DePalma et al^7^.


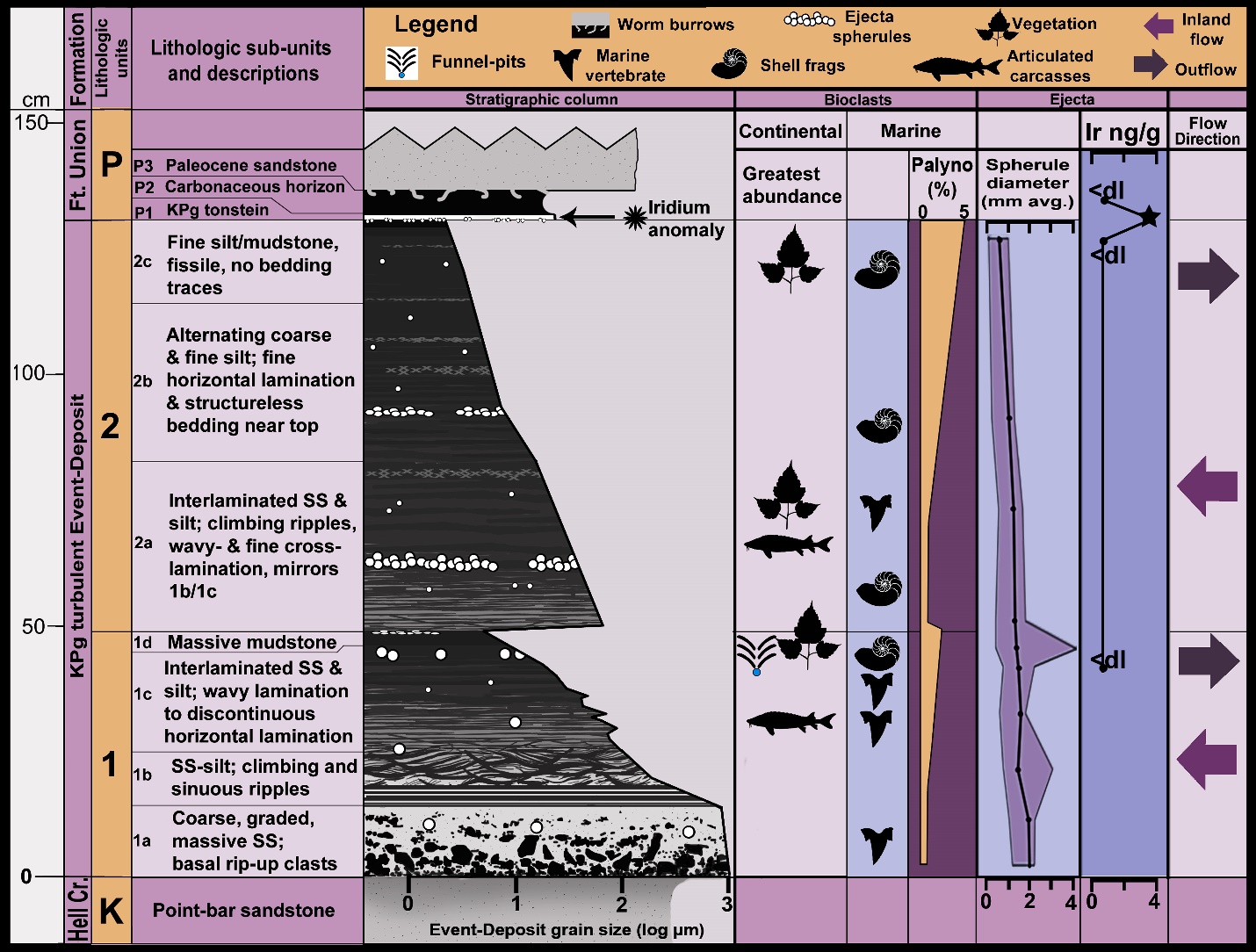


SUP MAT 3. **Histological sections of sturgeon fin spike FAU.DGS.ND.755.36.T.** (A), Histological section marking areas of interest, anterior carina oriented to left. (B and C), magnified views of the clearly defined growth bands and Lines of Arrested Growth (LAGs).


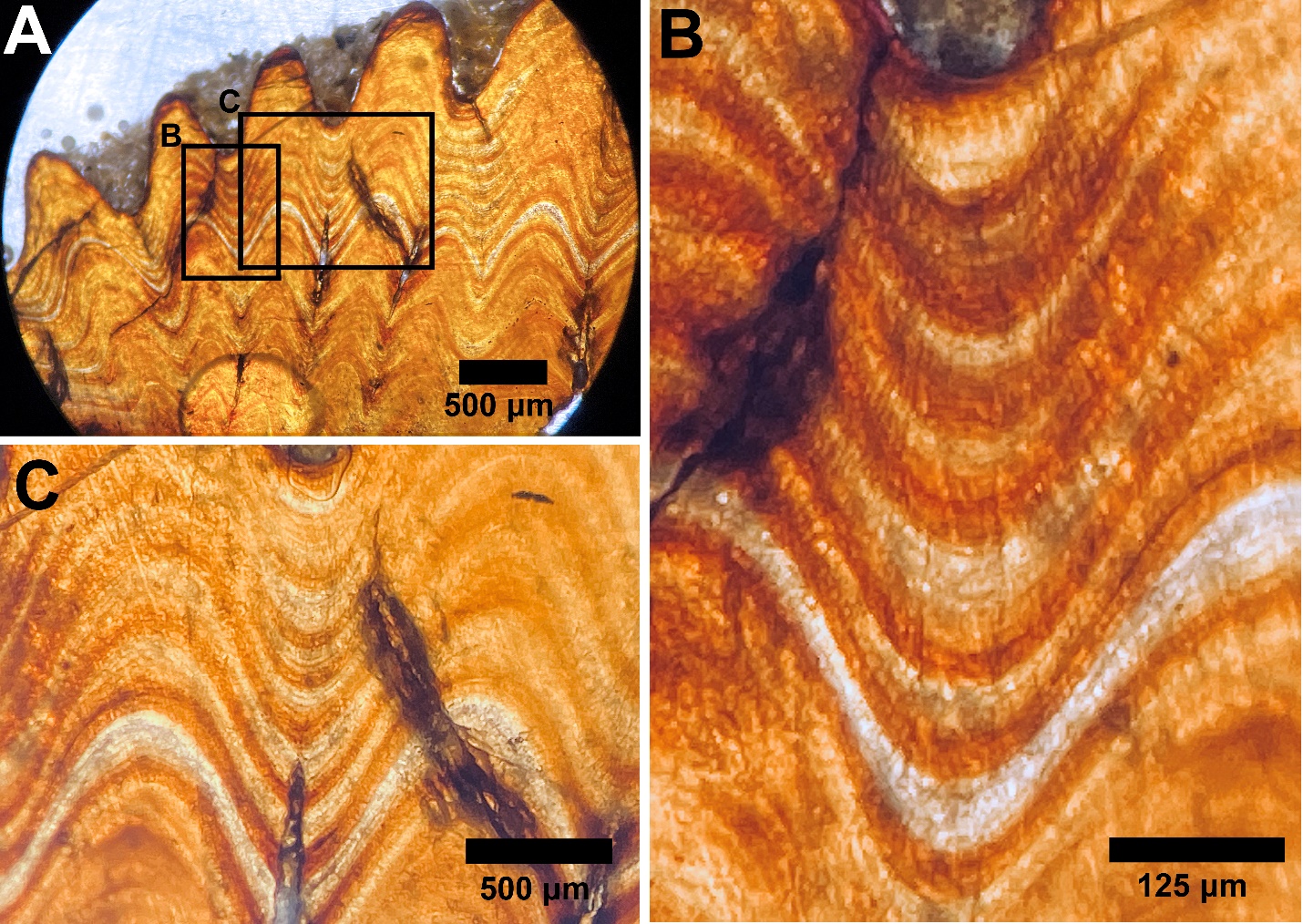


SUP MAT 4. **Histological sections of sturgeon fin spike FAU.DGS.ND.755.90.T.** (A), Cross-section of fin spike, anterior carina oriented to the right. (B), Magnified view showing clearly defined growth bands and LAGs.


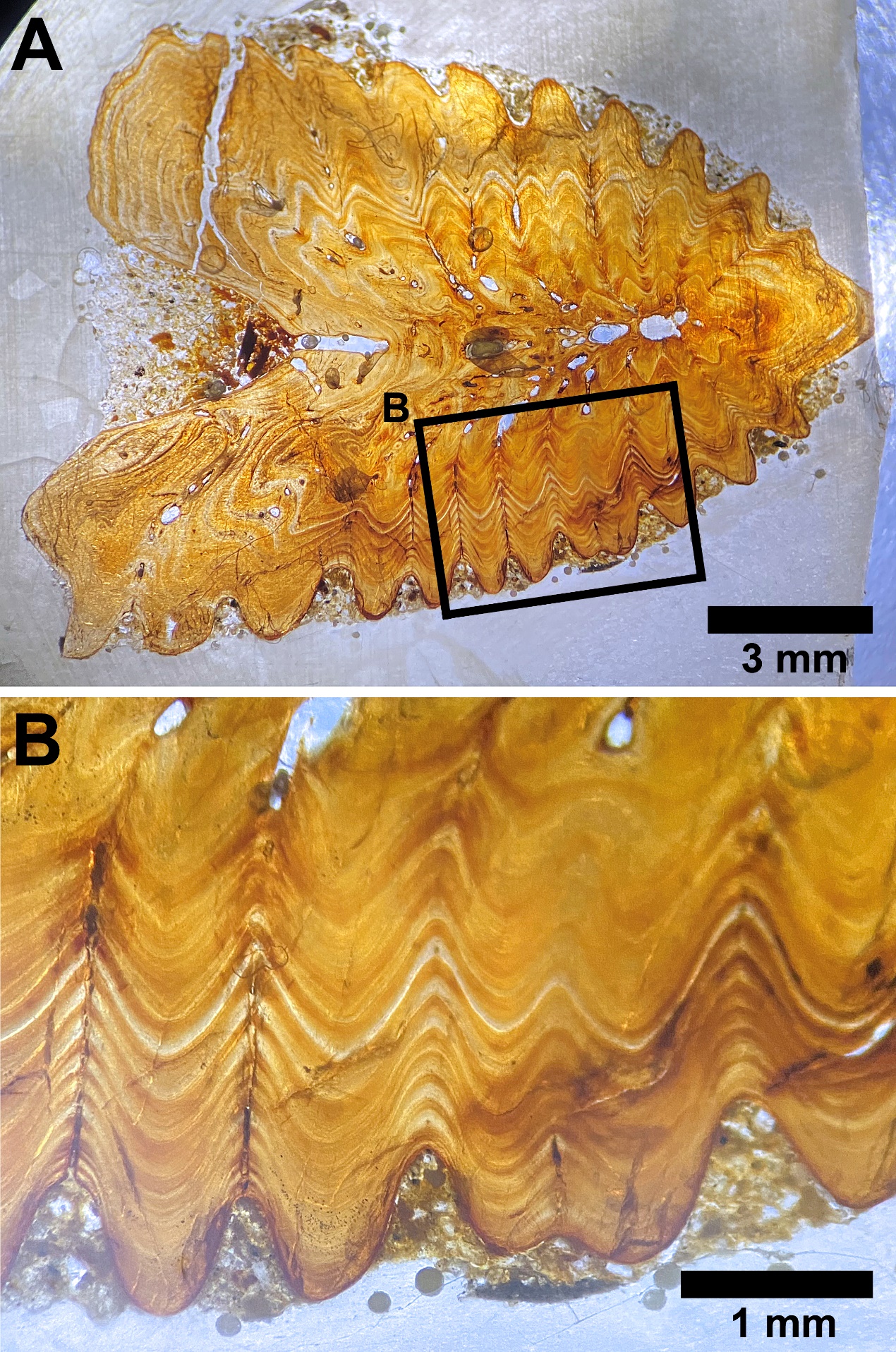


SUP MAT 5. **Histological sections of sturgeon fin spike FAU.DGS.ND.755.57.T.** (A), Histological section marking areas of interest, anterior carina oriented to the right. (B), magnified views of the clearly defined growth bands and Lines of Arrested Growth (LAGs).


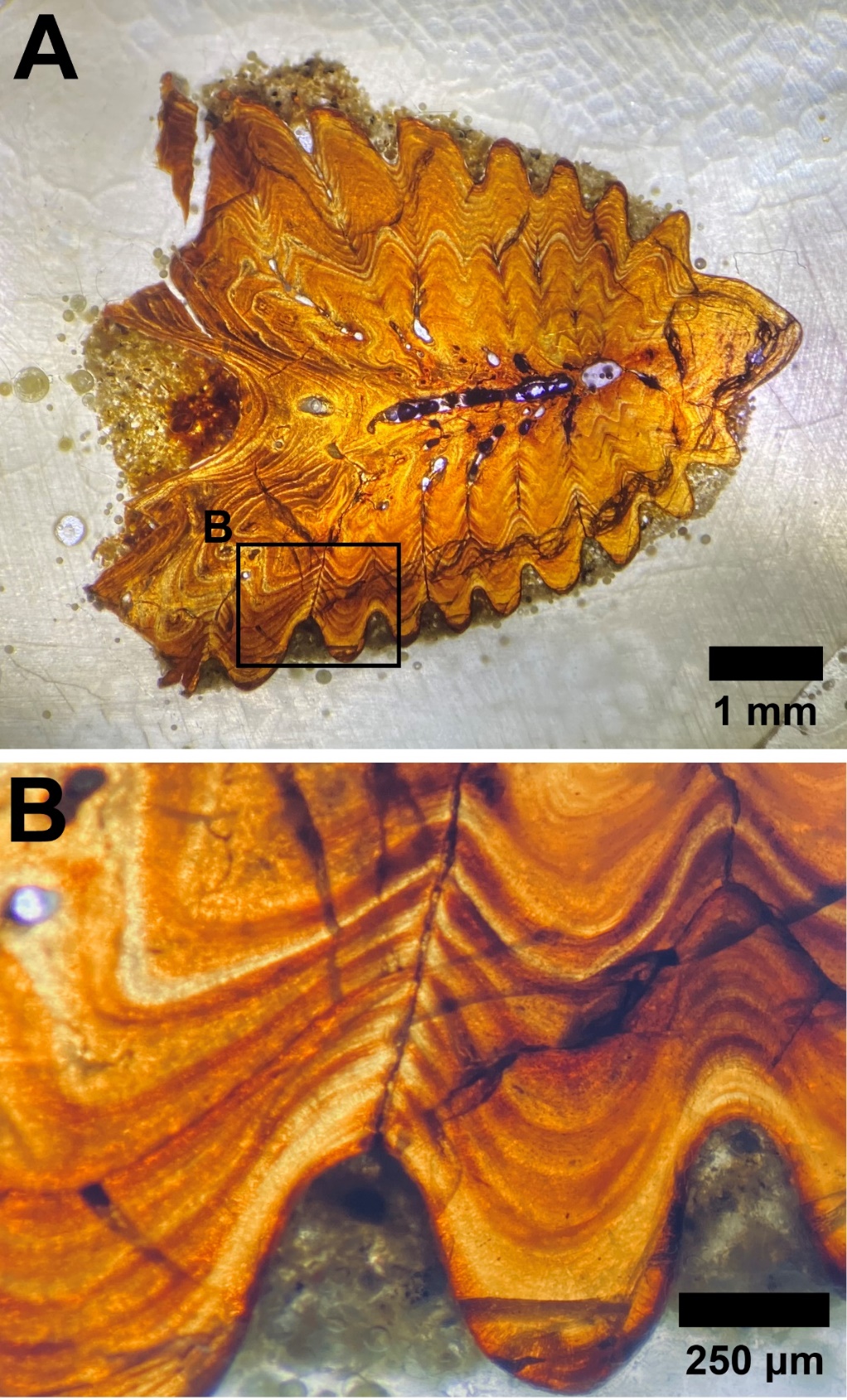


SUP MAT 6. **Histological section of paddlefish dentary FAU.DGS.ND.755.53.T.** (A), Cross-section of dentary, dorsal margin oriented to the right. (B), Magnified view showing clearly defined growth bands and LAGs.


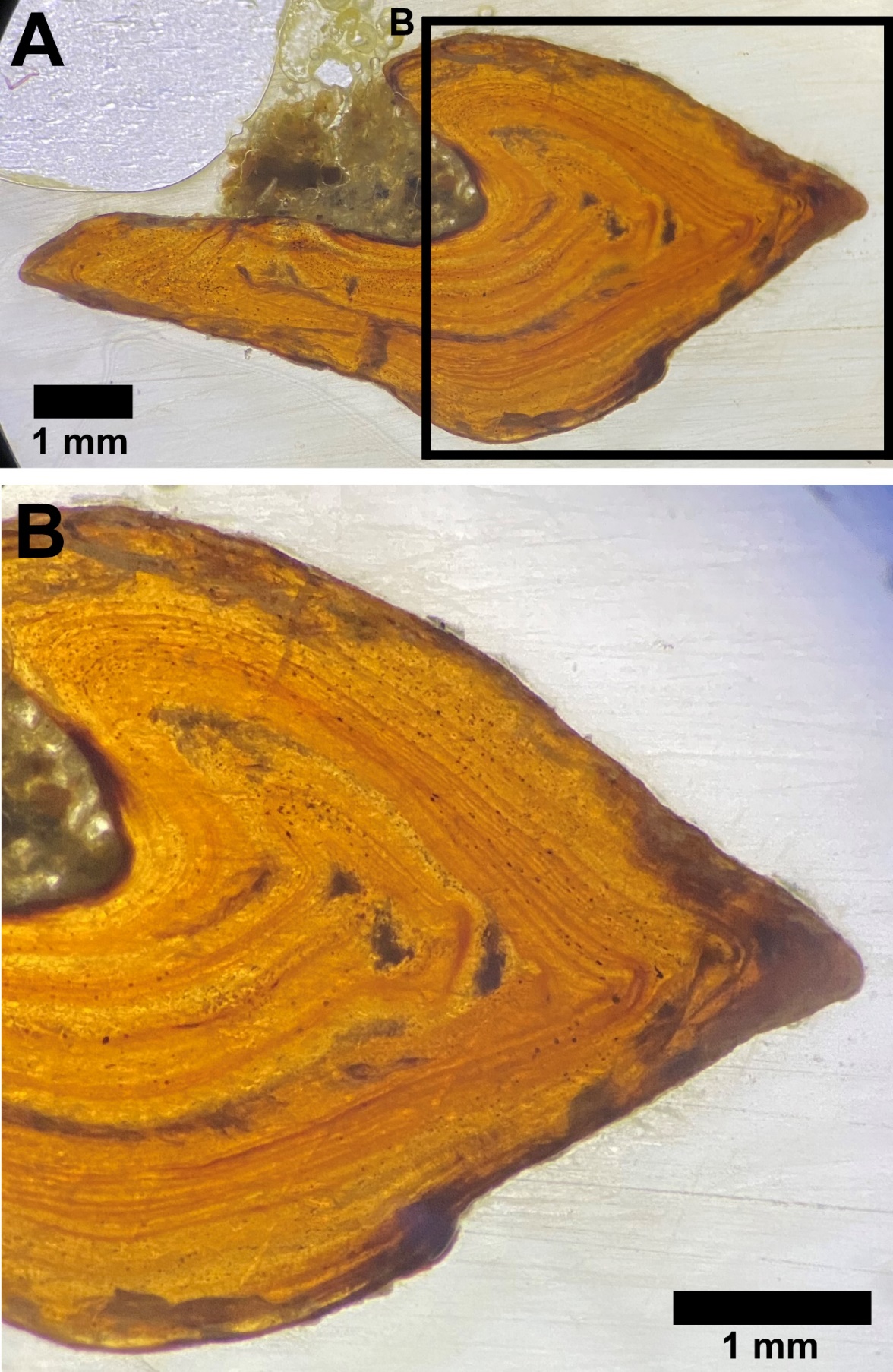


SUP MAT 7. **Histological section of paddlefish dentary FAU.DGS.ND.755.21.T.** (A), Cross-section of dentary, dorsal margin oriented to the right. (B), Magnified view showing clearly defined growth bands and LAGs.


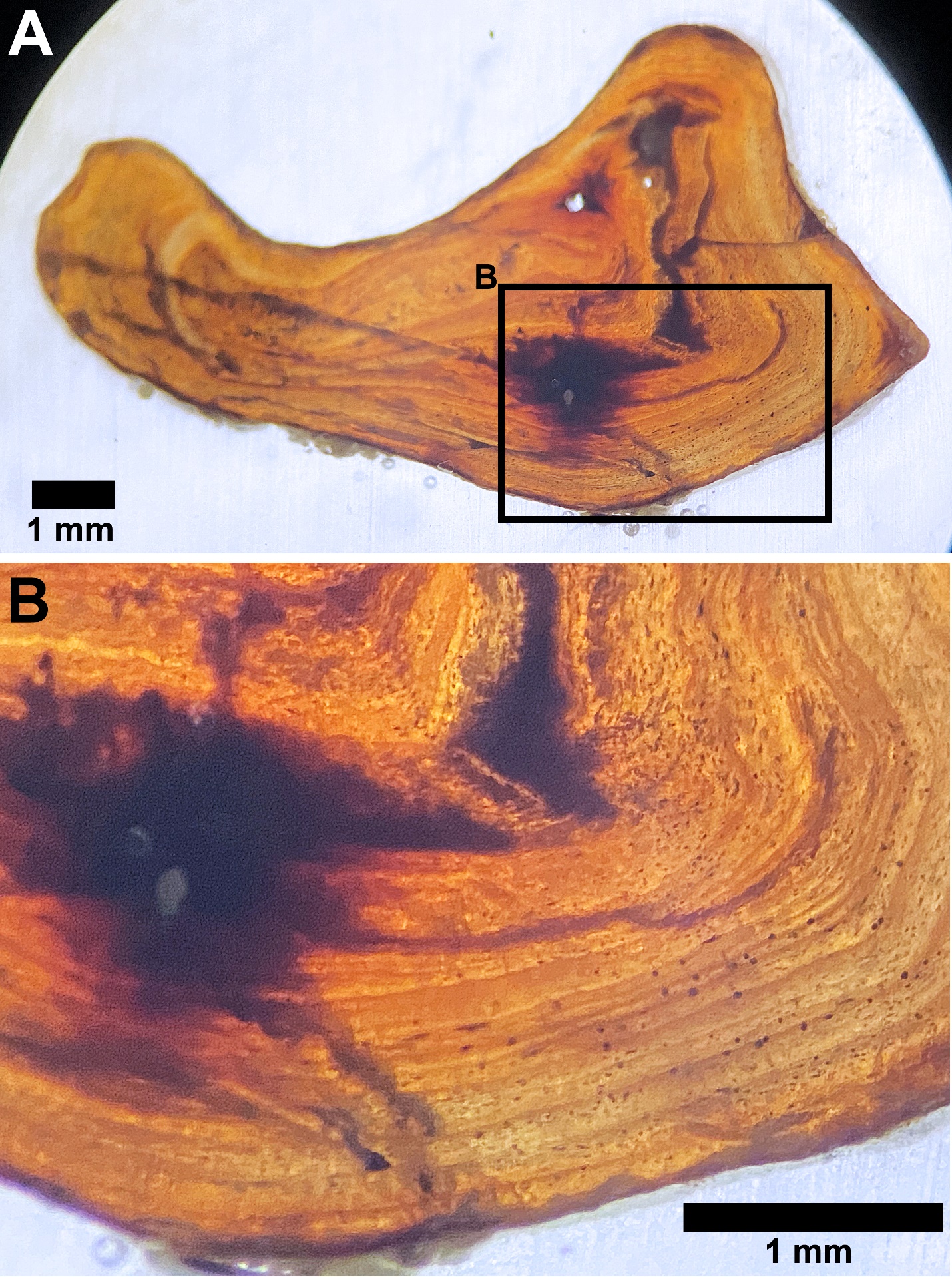


SUP MAT 8. **Histological section of paddlefish dentary FAU.DGS.ND.755.17.T.** (A), Cross-section of dentary, dorsal margin oriented to the left.


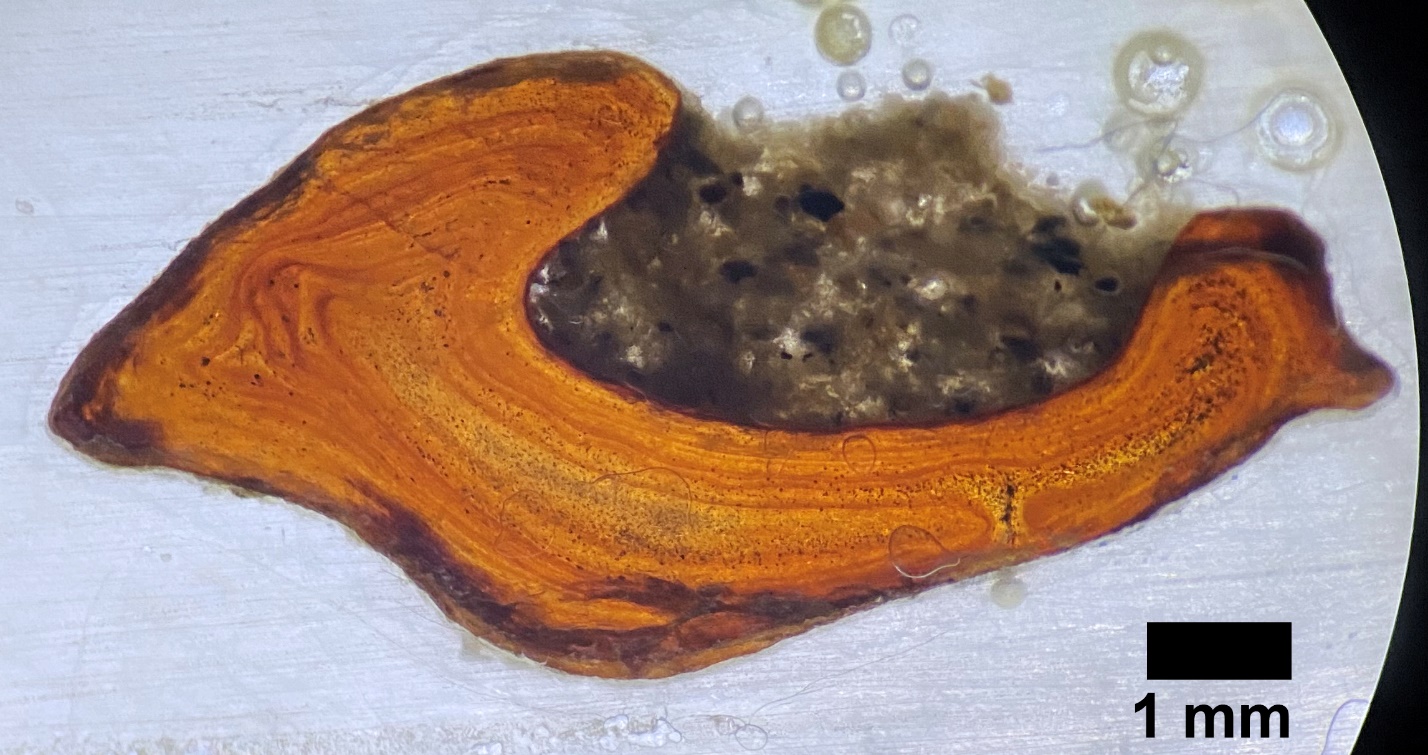


SUP MAT 9. **ẟ^18^O and ẟ^13^C data for sturgeon fin spikes, specimens 1 through 6.** ẟ^18^O and ẟ^13^C data shows repeated annual fluctuation, with dashed lines represent position of LAGs. Datum points marked by icons.


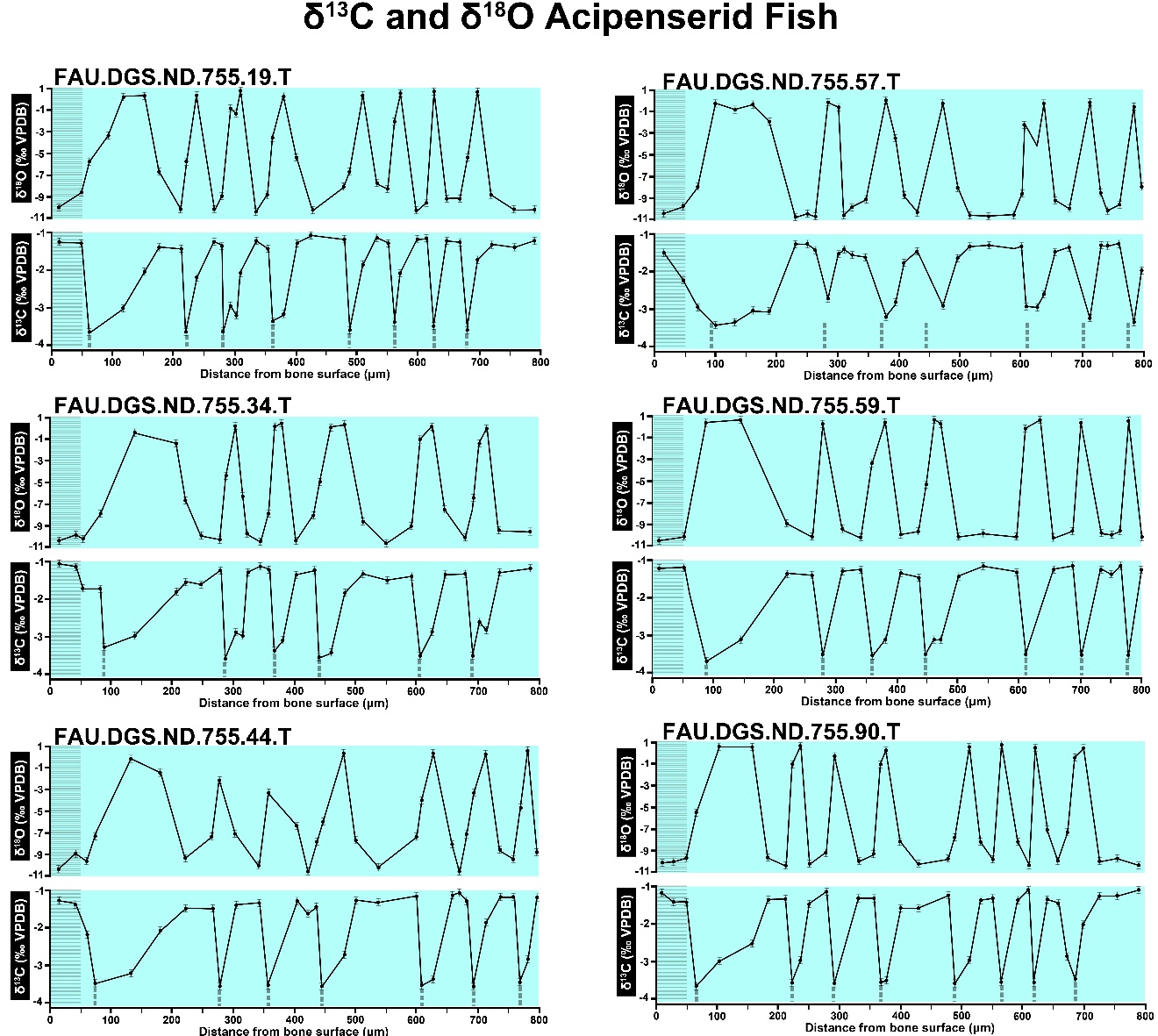


SUP MAT 10. **ẟ^18^O and ẟ^13^C data for sturgeon fin spikes, specimens 7 through 12.** ẟ^18^O and ẟ^13^C data shows repeated annual fluctuation, with dashed lines represent position of LAGs. Datum points marked by icons.


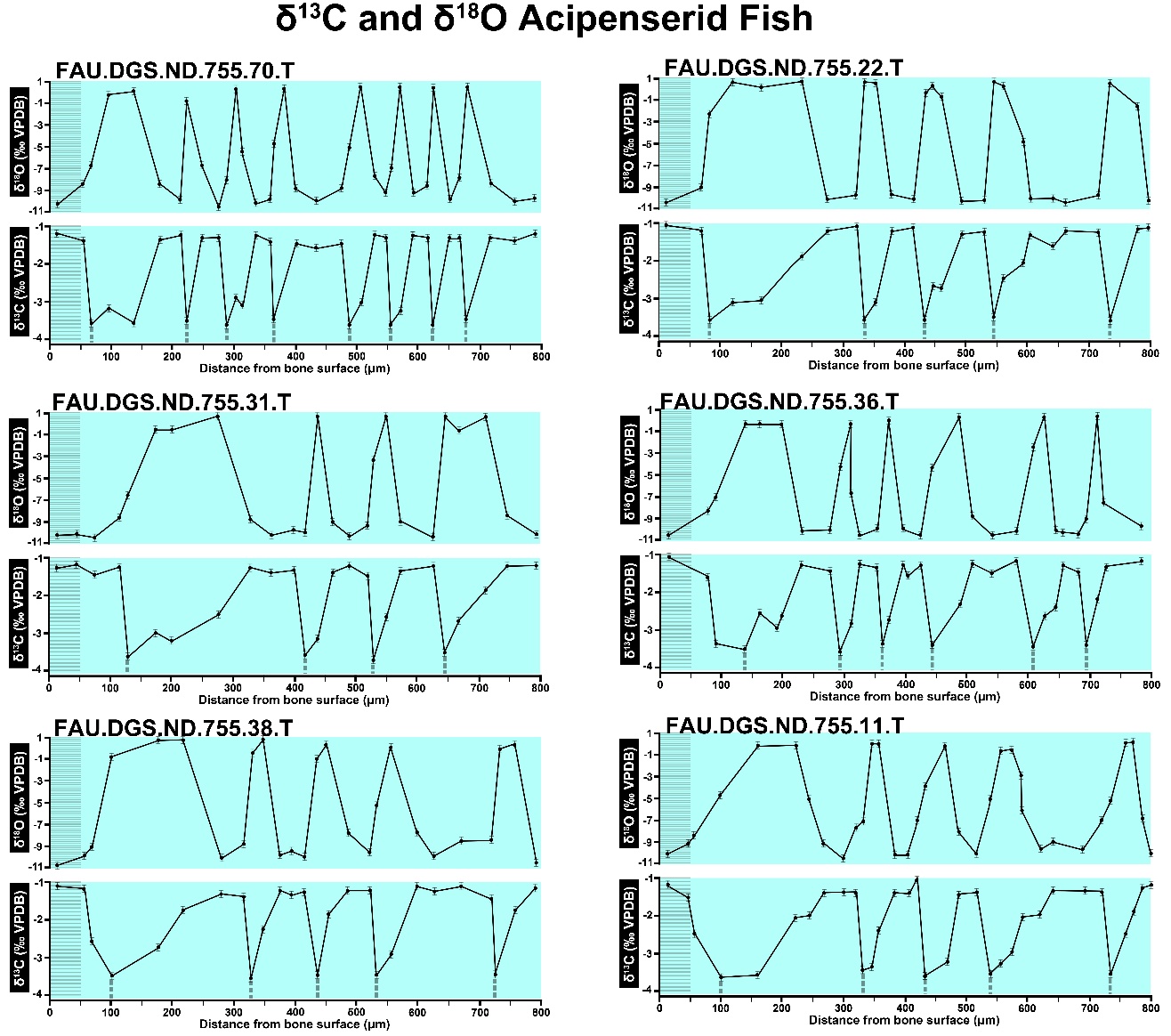


SUP MAT 11. **ẟ^18^O and ẟ^13^C data for paddlefish dentaries, specimens 1 through 7.** ẟ^13^C data shows repeated annual fluctuation; dashed lines represent position of LAGs. Datum points marked by icons.


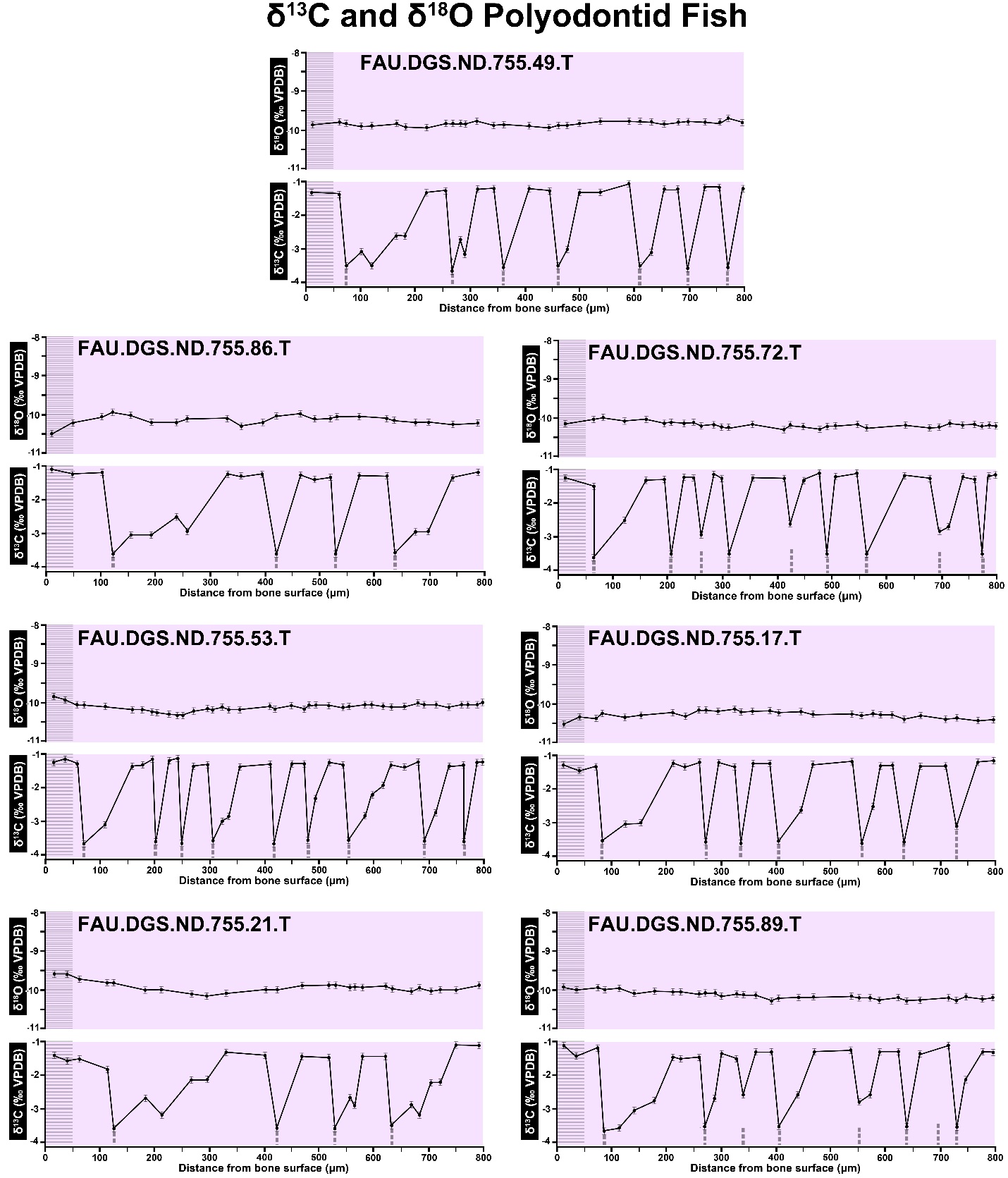


SUP MAT 12. **Superimposed ẟ^18^O and ẟ^13^C data for sturgeon fin spikes and paddlefish dentaries, calibrated to LAG spacing.** ẟ^18^O and ẟ^13^C data for acipenserids (sturgeon) shows a clear annual fluctuation that correlates with the ẟ^13^C data for the polyodontids (paddlefish). Datum points marked by icons, red arrows mark the position of LAGs.


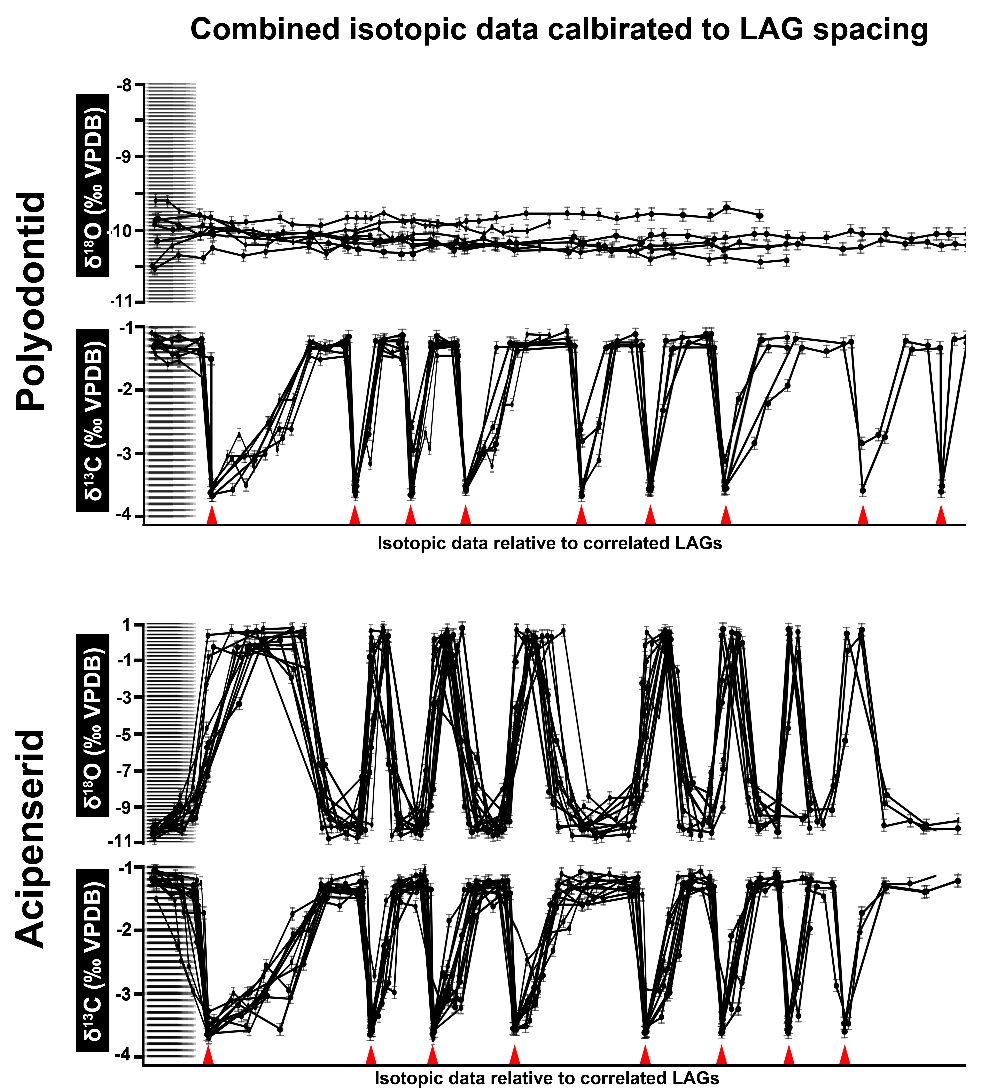


SUP MAT 13. **Representative leaf mining on angiosperm leaves.** (A-C), Serpentine leaf mines following the main leaf veins. A: FAU.DGS.ND.722.37.T; B: FAU.DGS.ND.722.42.T; C: FAU.DGS.ND.722.29.T.


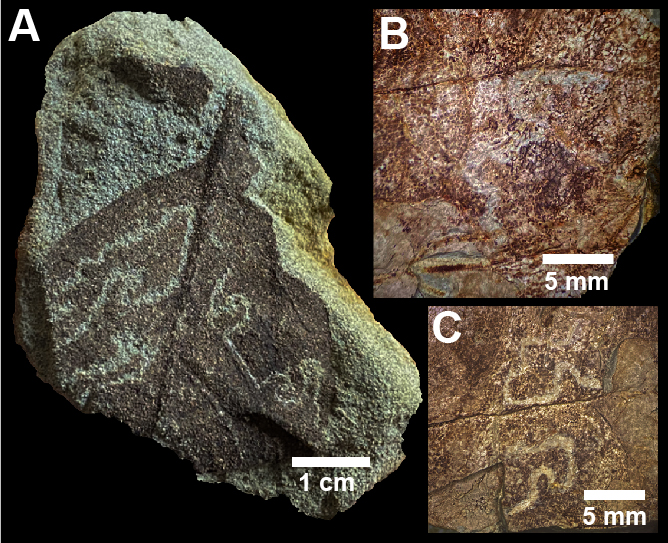


SUP MAT 14. **Mayfly burrow casts preserved in ironstone (FAU.DGS.ND.722.49.T).** (A), ventral view, and (B), lateral view of the U-shaped tunnels. Insert in (B) shows a cross-section of a modern analog.


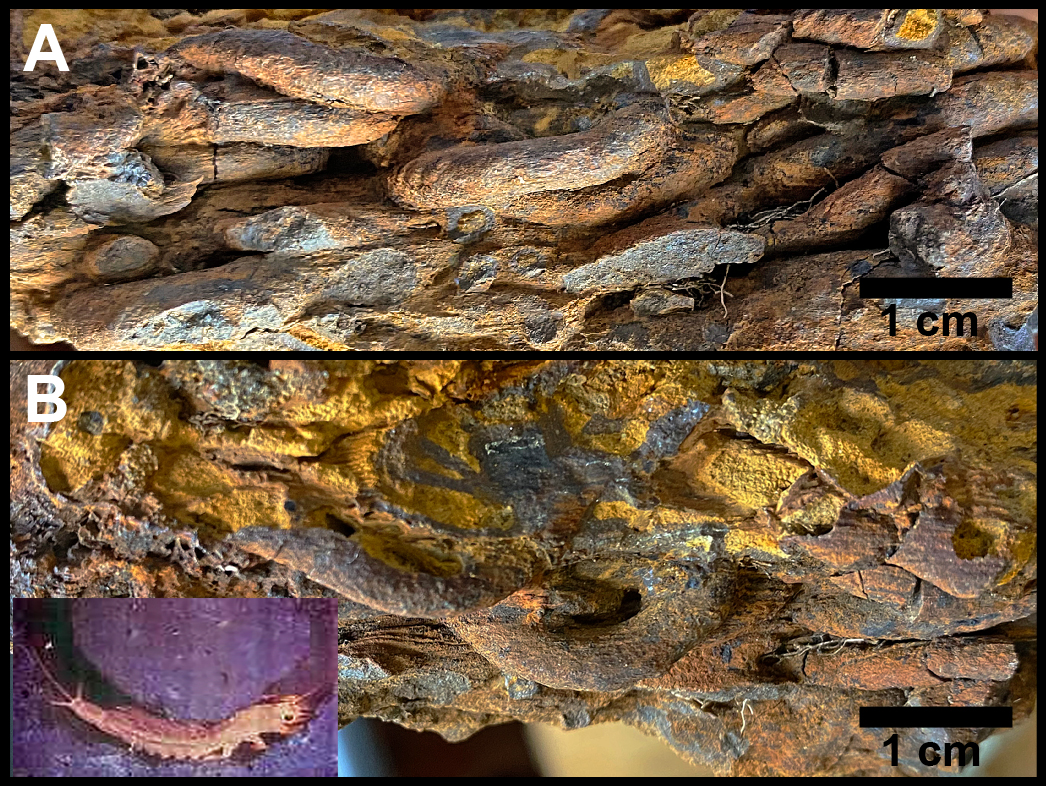


SUP MAT 15. **Mayfly burrow and body fossils.** (A), cross-section of mayfly burrow cast with preserved nymph inside (FAU.DGS.ND.743.22.T). (B), magnified view of abdominal section of nymph in (A), showing articulated sternite and tergite abdominal plates (arrows). (C, D), body fossils of adult mayflies from the Tanis Event-deposit. C: FAU.DGS.ND.743.39.T; D: FAU.DGS.ND.743.67.T.


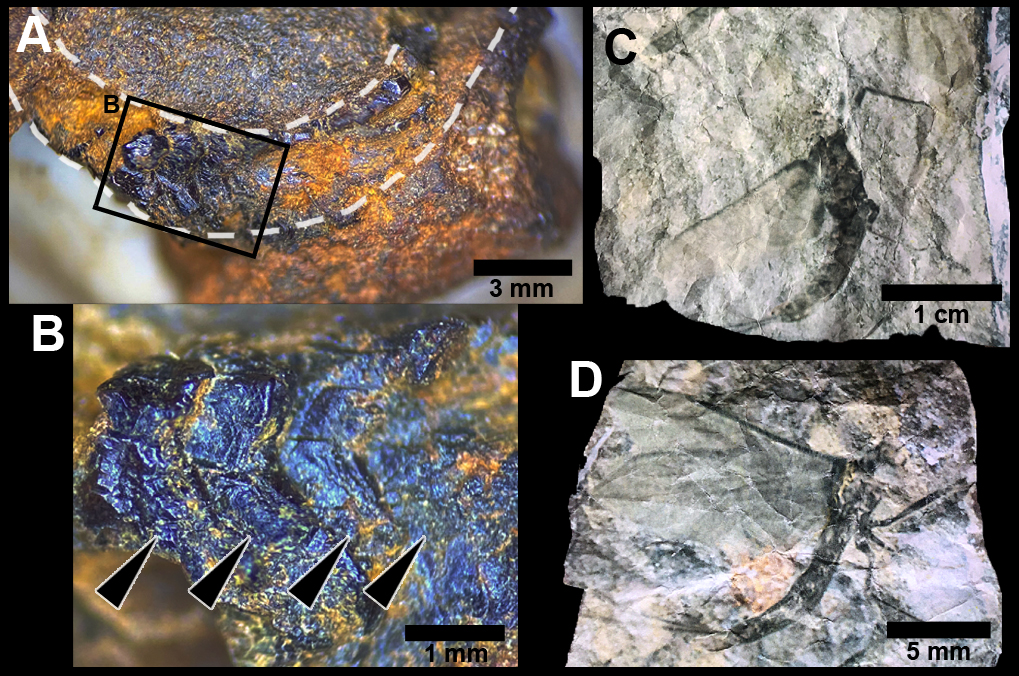

Supplement: Supplementary file 1 — Supplementary Information. [file 41598_2021_3232_MOESM1_ESM.docx]
